# Research on rehabilitation robot control based on port-Hamiltonian systems and fatigue dissipation port compensation

**DOI:** 10.3389/fbioe.2025.1609548

**Published:** 2025-05-23

**Authors:** Jingjing Li, Zhen Chen, Jian Li, Hongyu Yan, Zhen Li, Minshan Feng, Jiawen Zhan, Liwei Shao

**Affiliations:** ^1^ School of Automation, Beijing Institute of Technology, Beijing, China; ^2^ Greater Bay Area Innovation Institute, Beijing Institute of Technology, Zhuhai, China; ^3^ Wangjing Hospital, China Academy of Chinese Medical Sciences, Beijing, China; ^4^ Beijing Key Laboratory of Digital Intelligence Traditional Chinese Medicine for Preventing and Treating Degenerative Bone and Joint Diseases, Beijing, China

**Keywords:** upper-limb rehabilitation robot, muscle fatigue modeling, port-Hamiltonian system, passivity-based control, human-robot interaction stability

## Abstract

**Introduction:**

Upper-limb rehabilitation robots have been demonstrated to effectively promote motor recovery in stroke patients. However, in active training modes, control instability may be induced by the nonlinear and time-varying characteristics of muscle fatigue, increasing the risks of physical human-robot interaction and ultimately limiting rehabilitation outcomes.

**Methods:**

A novel control strategy within the port-Hamiltonian framework, incorporating a dynamic muscle fatigue model. Fatigue levels were assessed in real time using surface electromyography (sEMG) signals and mapped to damping parameters in joint space, enabling the port-based modeling of fatigue-related energy dissipation. A hierarchical control architecture was constructed, consisting of outer-loop admittance control and inner-loop energy shaping.

**Results:**

Theoretical analysis confirmed that the closed-loop passivity of the system was preserved and stability was ensured. Experimental validation further showed that, compared to fixed damping parameters, the proposed fatigue compensation approach reduced muscle fatigue accumulation by 45% and increased training duration by 40%.

**Discussion:**

The proposed fatigue-adaptive control framework was shown to enhance the safety, effectiveness, and physiological adaptability of rehabilitation training. The integration of real-time sEMG feedback and port-Hamiltonian modeling offers a promising solution for personalized robotic rehabilitation.

## 1 Introduction

Upper-limb rehabilitation robots have been widely applied in motor function restoration training for patients with conditions such as stroke (Ai et al., 2023). By providing highly repetitive and quantitatively controlled movement training, these technologies significantly enhance patients’ active participation and rehabilitation outcomes (Mahfouz et al., 2024; Xu et al., 2024). In active training modes, patients are required to perform movements independently, with the robot offering assistance only when necessary. However, as training sessions progress, muscle fatigue gradually accumulates, leading to decreased force output, reduced motion accuracy, and in some cases, early termination of training, which seriously undermines rehabilitation effectiveness (Thacham Poyil et al., 2020). If fatigue accumulation is not promptly identified and addressed during training, patients may struggle to complete the prescribed movements and even risk musculoskeletal injury (Groothuis et al., 2018). Moreover, patients often exert excessive force to meet training goals, which further accelerates fatigue development and increases the likelihood of injury. Therefore, real-time monitoring and compensation for patient fatigue has become a critical challenge for ensuring both the safety and effectiveness of robotic rehabilitation training.

In the field of robot-assisted rehabilitation, existing studies have combined biological signals with adaptive control to adjust robot parameters during the training process. For example, some studies use electromyography (EMG) signals to monitor the fatigue state of patients, adjusting virtual damping or stiffness coefficients in the early stages of fatigue to make robot movements smoother (Mashayekhi and Moghaddam, 2022). This EMG-based adaptive admittance control has shown better motion smoothness and accuracy in experiments compared to fixed-parameter control. In addition, some assistive control strategies adjust the control mode only when fatigue reaches a certain threshold through simple switching (Ghajari et al., 2023). This assist-as-needed strategy has also been effective in encouraging active patient participation (Lai et al., 2018; Cai et al., 2024).

However, despite the improvements these methods have made to training effectiveness and safety, they still have certain limitations. Current research mainly focuses on addressing fatigue states through parameter adjustment, but lacks a universal method for comprehensively modeling muscle fatigue from a system dynamics perspective. Existing control methods often overlook the principles of energy transfer and system stability, making it difficult to ensure passive stability in human-robot systems under different fatigue states. Muscle fatigue is nonlinear and time-varying. It involves not only the dissipation of local energy, but also changes in the global energy distribution and transfer mechanisms (Thacham Poyil et al., 2020; Groothuis et al., 2018). These challenges make accurate modeling and real-time control difficult.

To address this gap, the port-Hamiltonian System (PHS) provides an energy-based modeling method (Rashad et al., 2022). It effectively captures the dynamic characteristics of energy storage, dissipation, and exchange within a system. PHS also has the advantages of modularity and scalability (Rashad et al., 2019). It is not only suitable for describing complex dynamic systems but also facilitates the introduction of energy balance and passivity analysis in controller design (Groothuis et al., 2017). This provides theoretical support for addressing the nonlinear and time-varying issues caused by fatigue (Fujimoto et al., 2020; Sakata et al., 2024).

Based on the above analysis, this paper proposes a control method for upper-limb rehabilitation robots that integrates a muscle fatigue dynamic model. The goal is to address the limitations of existing control methods in fatigue compensation and dynamic adaptability by combining surface electromyography (sEMG) signals (Mashayekhi and Moghaddam, 2022; Vafadar et al., 2012) and port-Hamiltonian theory. The method models the patient’s muscle fatigue effect as a time-varying damping subsystem and evaluates fatigue levels in real-time. This allows muscle fatigue to be mapped as an additional dissipative element in the robot’s joint space, enabling dynamic modeling and response to fatigue states. This control strategy not only accurately tracks rehabilitation movements but also adjusts the robot’s assistive torque based on the real-time fatigue state, ensuring both safety and efficiency in rehabilitation training.

To overcome the limitations of existing methods in fully integrating fatigue states and energy transfer, the core objective of this study is to propose an innovative control framework. This framework is capable of dynamically and real-time responding to muscle fatigue, optimizing the relationship between robot assistive torque and fatigue compensation, thereby improving the safety and physiological adaptability of rehabilitation training. To achieve this, this paper proposes a passive controller based on energy shaping and damping injection (Sandoval et al., 2024; Kim et al., 2021). Additionally, by combining admittance control strategies (Liu et al., 2025), a dual-layer control system consisting of an outer and inner loop is constructed (Li et al., 2022). The outer loop adjusts the desired trajectory in real-time through admittance control, while the inner loop performs trajectory tracking and dynamically compensates for the additional damping introduced by fatigue, ensuring compliance and safety during the interaction. The main contributions of this paper are as follows.

(1) Fatigue Modeling: Muscle fatigue is modeled as additional dissipative elements in the joint space of the robot. As fatigue increases, greater damping is introduced into the joint dynamics. This effect is represented within the port-Hamiltonian framework by extending the joint damping matrix. A fatigue index is used to quantify the impact of fatigue on the system’s energy dissipation characteristics.
(2) Control Architecture: A dual-loop structure is proposed, with an outer admittance loop adjusting the desired trajectory based on interaction forces for compliance, and an inner loop ensuring accurate tracking while compensating for fatigue-induced damping. This balances compliance and tracking, resolving the stiffness-safety trade-off.
(3) Stability Guarantee: Based on the energy conservation principle of port-Hamiltonian systems and Lyapunov methods, it is proven that the closed-loop human-robot system, incorporating fatigue-related dissipation, remains strictly passive. This ensures the stability and safety of human-robot interaction.

The remainder of this paper is organized as follows. Section 2 presents the port-Hamiltonian modeling method of the system, the muscle fatigue model, and the control strategy design. Section 3 introduces the experimental setup and results. Section 4 provides analysis and discussion of the results. Section 5 concludes the paper.

## 2 Methods

Grounded in the port-Hamiltonian framework, this section introduces a novel formalization of the human–robot interaction system, wherein sEMG-based fatigue monitoring, an adaptive dissipation mechanism, and a dual-layer admittance–energy shaping control architecture are systematically integrated. This framework establishes a comprehensive rehabilitation training paradigm that simultaneously guarantees safety, compliance, and fatigue responsiveness, thereby laying a solid theoretical foundation for dynamic adaptation and individualized rehabilitation interventions.

### 2.1 Port Hamiltonian modeling of the system

To characterize the interaction between the rehabilitation robot and the human body from an energy perspective, this study adopts the port-controlled Hamiltonian (PCH) modeling approach. This method treats the mechanical system as an interconnection of atomic components such as inertial, elastic, and damping elements through ports, with a well-defined energy function description. For a typical upper-limb rehabilitation robot, its dynamic equations (Zhou et al., 2021) can be expressed in the joint space as:

$$M\left(q\right)\ddot{q}+C\left(q,\dot{q}\right)\dot{q}+G\left(q\right)+D_{0}\left(q\right)\dot{q}=\tau_{u}+\tau_{h}$$ (1)

Here, $q=\left(q_{1},q_{2},\ldots ,q_{n}\right)$ is the joint position vector. $M\left(q\right)$ is the positive definite inertia matrix. $C\left(q,\dot{q}\right)\dot{q}$ represents the Coriolis and centrifugal forces. $G\left(q\right)$ is the gravity term. $D_{0}\left(q\right)\dot{q}$ denotes the inherent joint damping or friction of the robot. $\tau_{u}$ is the control input torque. $\tau_{h}$ is the torque exerted by the patient on the robot joints, obtained by mapping the human-robot interaction force $F_{h}$ through the Jacobian transpose $J_{s}^{T}$ .

Equation 1 can then be rewritten in the port-Hamiltonian form (Sakata et al., 2024). In the port-Hamiltonian model, the Hamiltonian function, which represents the total energy of the system, is defined as:

$$H=\frac{1}{2}{\left(p\right)}^{T}M^{-1}\left(q\right)p+V\left(q\right)$$ (2)

where $p=M\left(q\right)\;\dot{q}$ are the generalized momenta, $V\left(q\right)$ is the potential energy. Then, an explicit port-Hamiltonian representation of the upper limb rehabilitation robot can be obtained by Equation 3.

$$\left(\begin{array}{l} \dot{q}\\ \dot{p} \end{array}\right)=\left(\underset{J\left(x\right)}{\underbrace{\left(\begin{array}{cc} 0_{n} & I_{n}\\ -I_{n} & 0_{n} \end{array}\right)}}-\underset{R\left(x\right)}{\underbrace{\left(\begin{array}{cc} 0_{n} & 0_{n}\\ 0_{n} & -D_{0}\left(q,p\right) \end{array}\right)}}\right)\left(\begin{array}{l} \nabla_{q}H\left(q,p\right)\\ \nabla_{p}H\left(q,p\right) \end{array}\right)+\left(\begin{array}{l} 0_{n}\\ G_{0}\left(q\right) \end{array}\right)\tau$$ (3)

Where $x={\left(q^{T},p^{T}\right)}^{T},$ ***J*** (***x***) is the 2*n* × 2*n* skew-symmetric structure matrix, defining the interconnection between efforts and flows, ***R*** (***x***) is also a 2*n* × 2*n* symmetric dissipative structure matrix. ***y*** is the output variables, and ***τ*** represents the external torque input, which comprises both the robot’s control input and the interactive torque exerted by the environment (the patient’s limb), as shown in Equation 4.

$$\tau =\tau_{u}+{J_{s}}^{T}F_{h}$$ (4)

Here, ***J***_*s*_ is the Jacobian in the inertial frame {0} (Lynch and Park, 2021), ***J***_*s*_^*T*^ is the transpose of ***J***_*s*_, ***F***_*h*_ is the contact force applied by the user to the end of the robot, which can be measured by the six-dimensional force sensor at the end of the robot. Note that $\langle {\left(\partial_{x}H\right)}^{T},\dot{x}\rangle$ is the power flow through the storage port, $\langle \tau ,\dot{q}\rangle$ is the power flow through the interaction port. The Dirac structure connects the ports by the structure matrix $J\left(x\right)$ .

### 2.2 Human-robot interaction

**FIGURE 1:**
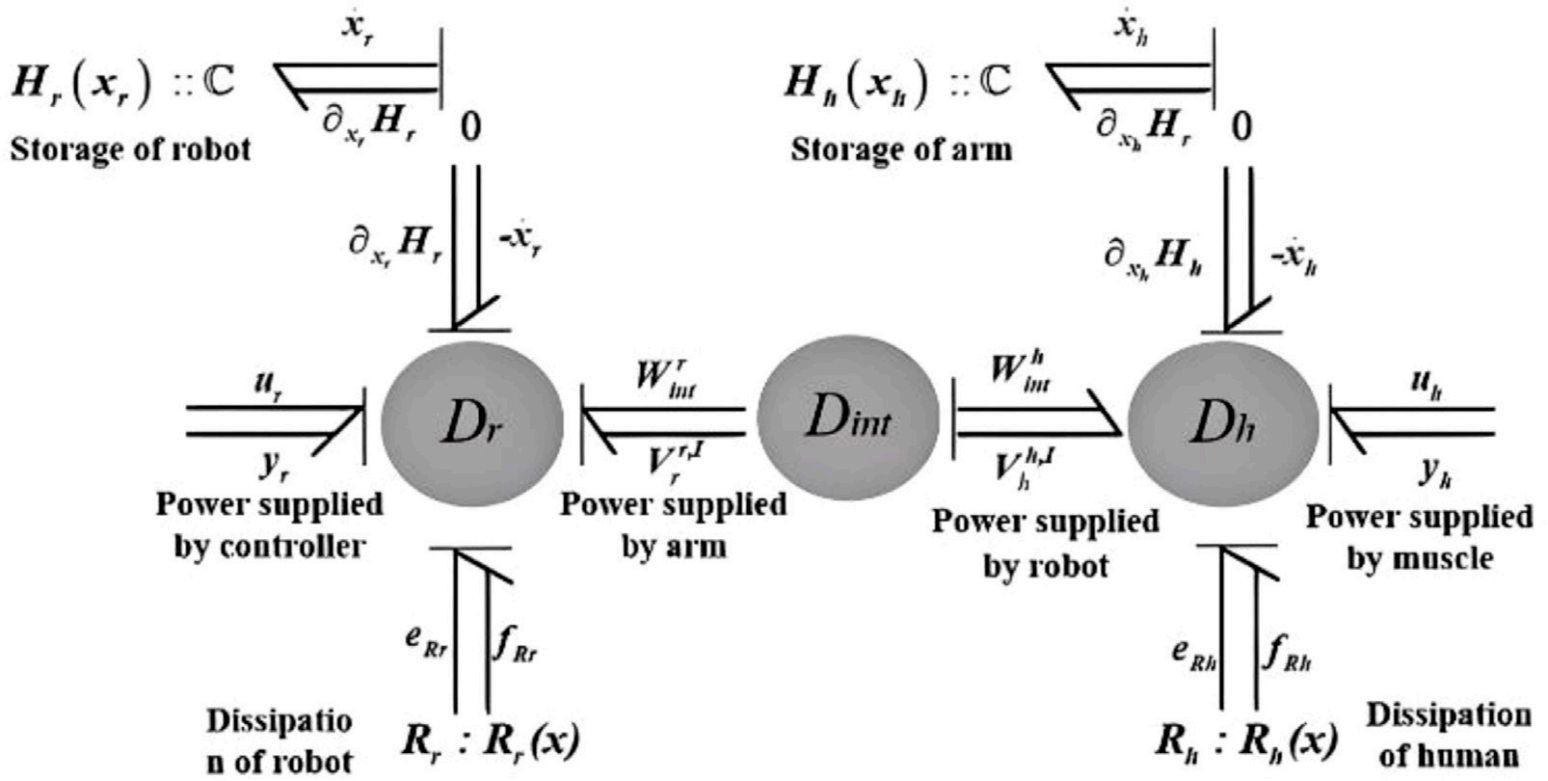
PH framework diagram for human-robot interaction.

During rehabilitation training, the continuous physical interaction is maintained between the patient and the robotic system through direct mechanical coupling. We consider the contact between the end of the upper limb rehabilitation robot and the hand as rigid contact in this paper. The interconnection structure of the robot and human based on the PH framework is shown in Figure 1.

In Figure 1, the element C represents the kinetic and potential energy stored by the robot, and the Dirac structure ***D*** represents the energy exchange between a certain subsystem and the outside world. ***D***_*r*_ denotes the rate of change of the total energy stored by the robot *H*_*r*_ equal to the power provided by resistive elements, the controller and arm, as formulated in Equation 5.

$${\dot{H}}_{r}={\left(u_{r}\right)}^{T}y_{r}+{\left(W_{\mathrm{int}}^{r}\right)}^{T}V_{r}^{r,I}+{\left(e_{Rr}\right)}^{T}f_{Rr}$$ (5)

***D***_*int*_ describes that the power supplied by the arm to the robot is equal to the negative of the power supplied by the robot to the arm, as expressed in Equation 6.

$${\left(W_{\mathrm{int}}^{r}\right)}^{T}V_{r}^{r,I}=-{\left(W_{\mathrm{int}}^{h}\right)}^{T}V_{h}^{h,I}$$ (6)

Here, V and W represent the twist and wrench, respectively. Within the framework of port-Hamiltonian systems, they are used to characterize the flow and effort variables associated with energy exchange between subsystems during human-robot interaction.

Based on power conservation property, the interconnection constraint between robot and arm is obtained as follows

$$\left(\begin{array}{l} W_{\mathrm{int}}^{r}\\ V_{h}^{h,I} \end{array}\right)=\left(\begin{array}{cc} 0 & Ad_{h_{r}^{h}}^{⊤}\\ -Ad_{h_{r}^{h}} & 0 \end{array}\right)\left(\begin{array}{l} V_{r}^{r,I}\\ W_{\mathrm{int}}^{h} \end{array}\right)$$ (7)

Note that considering two or more objects, that is, when there are multiple physical coordinate frames, the relative motion between them needs to be calculated and will have to be described in the same coordinate frame.

### 2.3 Fatigue dissipation port

During rehabilitation training assisted by an upper limb rehabilitation robot, patients are required to actively participate in completing the exercises (Ai et al., 2023). However, as the training progresses, their muscles gradually enter a state of fatigue. To ensure the safety and effectiveness of the rehabilitation process, it is essential to monitor and quantify the patients’ muscle fatigue in real time throughout the training session.

Muscle fatigue is characterized by energy dissipation and a reduction in output force (Wan et al., 2017). Based on the energy and port- Hamiltonian framework, muscle fatigue can be conceptualized as a dissipative port <Δ ***W***_*int*_^*h*^, ***V***_*h*_^*h*^>. Meanwhile, the characteristic parameter of electromyography (sEMG), Mean Power Frequency (MPF), consistently decreases during muscle fatigue (Zhang et al., 2024). As fatigue increases, the frequency components of the electromyographic signal change, with low-frequency components increasing and high-frequency components decreasing. By monitoring MPF changes, muscle fatigue levels can be assessed in real-time, and fatigue factors can be used to quantify its impact, providing scientific support for fatigue monitoring and adjustment. The advantage of MPF lies in its ability to reflect muscle fatigue in real-time, thereby optimizing robot control strategies and rehabilitation training outcomes. The fatigue factor *f*_*atigue*_ is defined as:

$$f_{atigue}=\frac{{\text{MPF}}_{init}-{\text{MPF}}_{current}}{{\text{MPF}}_{init}}$$ (8)

where $f_{atigue\;}\in \left[0,1\right],$ $f_{atigue\;}=0$ , indicates no fatigue, while $f_{atigue\;}=1$ represents extreme fatigue. And MPF_*int*_ and MPF_*current*_ are the original, and current MPF values of the sEMG signal, respectively. Then, Δ ***W***_*int*_^*h*^ can be expressed as:

$$\Delta W_{\mathrm{int}}^{h}=f_{atigue}\;K_{h}\;V_{h}^{h,0}$$ (9)

Here, $K_{h}\in R^{6\times 6}$ is a positive definite adjustment matrix.

Using Equations 7, 9, Equation 10 is obtained.

$$\Delta W_{\mathrm{int}}^{h}=f_{atigue}K_{h}Ad_{h_{r}^{h}}J_{s}\;{\dot{q}}_{r}$$ (10)

To prevent discomfort or secondary injuries caused by fatigue, rehabilitation robots must continuously monitor human-robot interaction forces and dynamically adjust their assistance level. By leveraging the port-Hamiltonian framework, real-time tuning of the system’s dissipation matrix enables precise control of energy flow, ensuring system stability while adaptively distributing assistive torque.

In the port-Hamiltonian system, the flow and dissipation of energy are described through multiple ports, with each port representing an energy exchange interface in the system. Specifically, the fatigue dissipation port is responsible for describing the energy loss caused by muscle fatigue, which directly affects the robot’s assistive torque adjustment. The introduction of the fatigue dissipation port enables dynamic adjustment based on real-time muscle states, ensuring system stability under different fatigue conditions. Furthermore, the port-Hamiltonian system connects various subsystems and ports through the Dirac structure. The Dirac structure describes the inherent principles of energy exchange and transfer. Building on this concept, the dissipation matrix of the rehabilitation robot can be expressed in Equation 11.

$$D_{tol}\left(x,f_{atigue}\right)=D\left(x\right)+\gamma D_{f}\left(x,f_{atigue}\right)$$ (11)

Here, γ>0 is a design parameter used to regulate the compensatory torque, which can be flexibly configured according to the rehabilitation needs of different patients. Meanwhile, $D_{f}\left(x,f_{atigue}\right)$ is a positive (or positive semi-definite) matrix that provides “additional dissipation” when patient fatigue intensifies. From a control perspective, this design allows the robot to inject greater damping or assistive force, thereby partially taking over the patient’s training effort. As a result, the robot introduces the compensation dissipation port < ***τ***_*comp*_, ***q̇*** > to compensate for the decrease of muscle force due to fatigue. Hence, Equation 12 is derived.

$$\tau_{comp}=\gamma D_{f}\left(x\right)\;\dot{q}$$ (12)

Based on the PH modeling method, all subsystems are interconnected through the Dirac structure. The power conserving property of the Dirac structure ensures that the energy change in the additional compensatory dissipative port introduced by the robot equals the power dissipated by the muscle due to fatigue, and this relationship is expressed in Equation 13.

ΔWinthVhh,IT=τcompTq˙ (13)

By combining the analytical expressions from Equations 15, 19, Equation 14 is obtained.

$$D_{f}\left(x,f_{atigue}\right)=\frac{f_{\text{atigue}}}{\gamma }J_{s}^{T}K_{h}Ad_{h_{r}^{h}}J_{s}$$ (14)

### 2.4 Feature extraction of sEMG signals

EMG signals have significant applications in rehabilitation medicine and motor control (Tian et al., 2024), as they sensitively reflect muscle activity and the regulatory state of the nervous system. Essentially, EMG signals represent the superimposed action potentials generated by muscle fibers. By thoroughly analyzing various EMG signal characteristics, it is possible to identify physiological phenomena such as muscle fatigue, muscle synergy, and movement intentions. For example, when muscle fatigue occurs, frequency-domain characteristics of the EMG signals, such as the mean power frequency (MPF) and median frequency (MDF), decrease notably, while time-domain features, such as the root mean square amplitude (RMS), typically increase or fluctuate. Real-time monitoring and analysis of these variations allow accurate evaluation of muscle fatigue, thus enabling dynamic adjustment of rehabilitation training intensity to prevent potential damage caused by excessive fatigue. Therefore, in-depth analysis and effective utilization of EMG signals, especially fatigue-related features, are crucial for improving the safety, effectiveness, and personalization of rehabilitation training.

**FIGURE 2:**
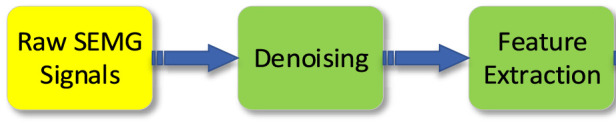
sEMG signal processing.

In practical measurement and analysis, sEMG as a non-invasive type of EMG signal, has been widely employed in clinical rehabilitation and movement assessment (Tian et al., 2024). sEMG signals effectively reveal underlying patterns associated with muscle fatigue (Zhang et al., 2024). However, raw sEMG signals generally contain noise and interference, necessitating preprocessing and feature extraction procedures to ensure accurate subsequent analysis. Typically, the sEMG signal processing workflow primarily involves signal denoising and feature extraction, as illustrated in Figure 2.

In this study, sEMG signals obtained from the MYO armband were already initially preprocessed using its embedded algorithms. Therefore, this research focuses specifically on feature extraction from sEMG signals for estimating muscle fatigue. Previous studies have widely recognized that MPF progressively decreases as muscle fatigue intensifies (Thacham Poyil et al., 2020). Consequently, this research adopts MPF as a key feature parameter to effectively evaluate muscle fatigue, facilitating precise assessment and dynamic adjustment of rehabilitation training.

### 2.5 Control architecture

**FIGURE 3:**
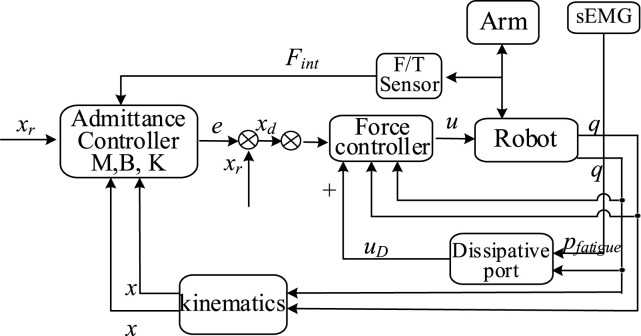
Human-robot interaction control block diagram.

To balance compliant adaptation to patient intent and precise trajectory tracking, a dual-layer control architecture is proposed. In this framework, the desired trajectory is adjusted in real time by the outer admittance control layer based on interaction forces, while accurate tracking and compensation for fatigue-induced dynamic variations are ensured by the inner layer. Through this coordinated structure, both compliance and safety are achieved (see Figure 3).

The outer-loop admittance control (Li et al., 2022), in error form, is written as

$$M_{a}\ddot{e}+B_{a}\dot{e}+K_{a}e=F_{h}$$ (15)

Where $e\in R^{m}$ is the admittance error, $M_{a}$ , $B_{a}$ , $K_{a}\in R^{m\times m}$ are inertia, damping and stiffness parameters of impedance control, respectively, and ***F***_*h*_ is the contact force applied by the user to the end of the robot. The corrected desired trajectory $x_{d}$ is obtained via $x_{d}=x_{r}+e$ . Here, $x_{r}$ denotes an original desired trajectory of the robot. From Equation 15, it can be seen that if the patient applies a force $F_{h}$ , the error e will change accordingly, thereby modifying $x_{r}$ into $x_{d}$ ; if the patient is unable to apply any force (i.e., $F_{h}=0$ ), Equation 15 converges to e=0 , indicating that $x_{d}\approx x_{r}$ . Therefore, Equation 15 represents the outer-loop compliance adjustment based on the human-machine interaction force.

In the inner-loop, our objective is to ensure that the robot’s actual end-effector position x precisely tracks the desired trajectory $x_{d}$ , while the joint space q follows the desired joint angles $q_{d}$ via inverse kinematics. Under the port-Hamiltonian framework, this goal is achieved by employing a method that combines energy shaping with damping injection. The newly introduced energy function $H_{e}$ is employed to modulate the system’s energy level and actively guide it toward the desired equilibrium. It is expressed in Equation 16.

$$H_{e}=V\left(q\right)+\frac{1}{2}{\left(q-q_{d}\right)}^{⊤}K_{p}\left(q-q_{d}\right)$$ (16)

Damping is introduced into the system by mapping the joint velocities $\dot{q}$ to $K_{v}\dot{q}$ , thereby realizing effective energy dissipation. Then, Equation 17 is formulated as follows.

$$\tau_{u}=-C\left(q,\dot{q}\right)\dot{q}-G\left(q\right)+\left(D_{0}\left(x\right)+\gamma D_{f}\left(x,f_{atigue}\right)\right)\dot{q}-K_{p}\left(q-q_{d}\right)-K_{v}\left(\dot{q}-{\dot{q}}_{d}\right)$$ (17)

To carry out a unified energy analysis, one can reconstruct Equation 15 within the port-Hamiltonian framework. We define the state variable $\left(e^{T},p_{e}^{T}\right)$ , so that the Hamiltonian (energy) for the subsystem is given by the Equation 18.

$$H_{e}\left(e,p_{e}\right)=\frac{1}{2}p_{e}^{⊤}M_{a}^{-1}p_{e}+\frac{1}{2}e^{⊤}K_{a}e$$ (18)

Equation 15 can likewise be rewritten as

$$\underset{{\dot{x}}_{e}}{\left(\begin{array}{l} \dot{e}\\ {\dot{p}}_{e} \end{array}\right)}=\underset{J_{e}}{\underbrace{\left(\begin{array}{cc} 0 & I\\ -I & 0 \end{array}\right)}}\left(\begin{array}{l} \nabla_{e}H_{e}\left(e,p_{e}\right)\\ \nabla_{p_{e}}H_{e}\left(e,p_{e}\right) \end{array}\right)-\underset{R_{e}}{\underbrace{\left(\begin{array}{cc} 0 & 0\\ 0 & B_{a} \end{array}\right)}}\left(\begin{array}{l} \nabla_{e}H_{e}\left(e,p_{e}\right)\\ \nabla_{p_{e}}H_{e}\left(e,p_{e}\right) \end{array}\right)+\underset{G_{e}}{\left(\begin{array}{l} 0\\ I \end{array}\right)}F_{h}$$ (19)

Among these, $R_{e}$ corresponds to $B_{a}$ , $J_{e}$ is a typical skew-symmetric structural matrix (in the position–momentum coordinates), and $F_{h}$ is the external force input port. It can be seen that this is a port-Hamiltonian system with damping (with the resistive element $R_{e}$ ), exhibiting passivity at the external force port $F_{h}$ .

The system now comprises two port-Hamiltonian subsystems. The first is the robot body and inner-loop control subsystem, where the end-effector force input and output velocity are coupled with the external force $F_{h}$ through a port. The second is the outer-loop admittance subsystem, which takes $F_{h}$ as its input and produces the error state ( $e,\dot{e}$ ), thereby determining the desired pose $x_{d}$ .

These two subsystems interact with each other via the same force $F_{h}$ ; that is, the patient applies a force at the robot’s end-effector, the robot senses $F_{h}$ , and the outer-loop system uses $F_{h}$ as its driving input. Under ideal zero-delay and perfect sensing conditions, they are interconnected passively at the port, meaning that they merely exchange power without generating or consuming any extra energy (aside from the inherent damping loss). Due to the passivity of single-port Hamiltonian systems, when multiple systems are interconnected, the resulting overall port-Hamiltonian system still preserves the property of energy being only conserved or dissipated. This ensures stability throughout rehabilitation training, preventing instability or abnormal energy release, and effectively safeguarding user safety.

## 3 Results

### 3.1 Experimental setup

**FIGURE 4:**
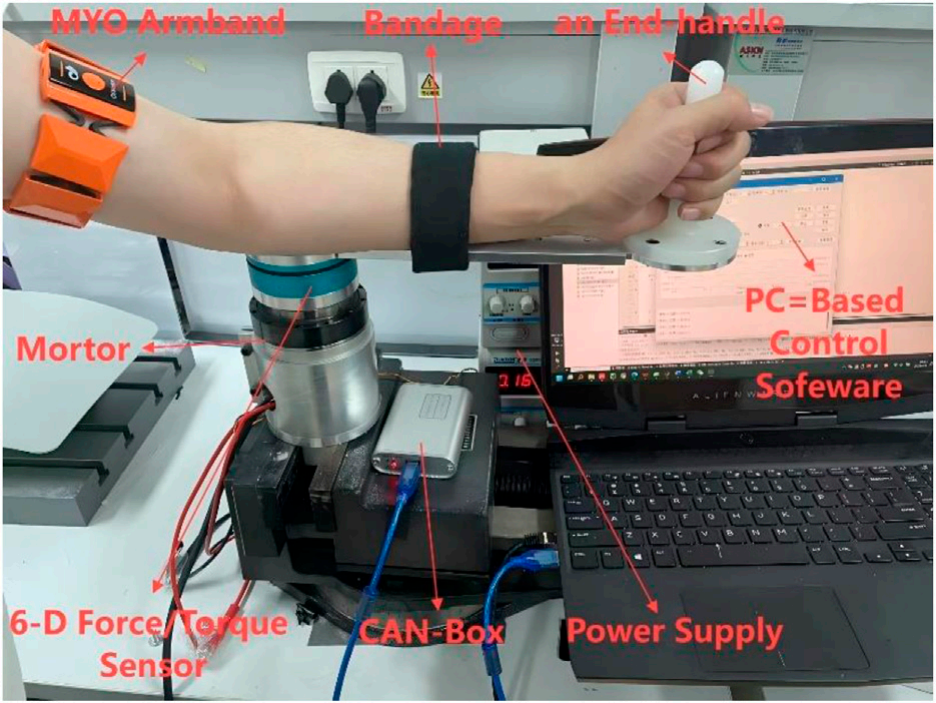
Schematic diagram of the experimental platform.

A self-developed single-degree-of-freedom upper limb rehabilitation device was used in this experiment to perform active and passive elbow flexion-extension training. The device consists of a motor-driven rotational joint with a handle at the end for the subject to hold. During training, the subject’s upper arm is secured with straps while performing flexion and extension movements. The system integrates a high-resolution encoder to measure elbow joint angles in real time and a six-axis force sensor at the joint to capture human-robot interaction forces. To monitor muscle fatigue, surface electromyography (sEMG) signals are collected from the biceps and triceps using MYO armbands. The overall structure of the device is shown in Figure 4.

In this study, the training task was designed as repetitive flexion and extension of the elbow joint. Participants were instructed to follow a predefined rhythm and track a sinusoidal target trajectory ranging from 0° to 90°, performing forearm movements as synchronously as possible. Each participant underwent training under two control strategies: (A) Fixed-parameter control, in which a dual-loop control structure was applied with constant control parameters throughout the session; and (B) Fatigue-compensated control, a dual-loop control method proposed in this study, which dynamically adjusted control parameters in real time based on the estimated muscle fatigue state. The training duration was not limited in advance and continued until the participant was no longer able to follow the rhythm or complete the required range of motion, which was defined as reaching fatigue failure. To ensure safety, the maximum duration of a single training session was set at 400 s, and participants could terminate the session at any time if they experienced discomfort. A rest period of at least 30 min was provided between the two control conditions to allow full recovery from muscle fatigue.

A total of 6 healthy, right-handed adult participants aged between 24 and 36 years, with no known neuromuscular disorders, were recruited for the experiment. The average age of the participants was 27.33 ± 4.97 years (mean ± SD). During the training sessions, surface electromyography (sEMG) signals were collected from the biceps brachii and triceps brachii using an armband device at a sampling frequency of 500 Hz. The acquired sEMG data were utilized for estimating muscle fatigue and for real-time adjustment of the control parameters. Training performance was evaluated based on muscle fatigue features, specifically the mean power frequency (MPF), as well as the total training duration. These measures were used to assess the effectiveness of the proposed control strategy in reducing fatigue and improving training stability.

### 3.2 Evaluation metric

#### 3.2.1 Muscle fatigue rate of change ( ${\dot{F}}_{atigue}$ )

Defined as the rate of increase in the muscle fatigue index per unit time, this metric is approximated by the difference between the pre- and post-training fatigue indices divided by the training duration (or up to the time of interruption, if the training was not completed). It quantifies the rate of fatigue accumulation, with lower values indicating slower buildup. The metric is expressed in Equation 20.

$${\dot{F}}_{atigue}=\frac{f_{atigue}\left(t_{end}\right)-f_{atigue}\left(t_{start}\right)}{T}$$ (20)

Here, $t_{start}$ , $t_{end}$ and T denote the start time, end time, and total duration of the rehabilitation training, respectively.

#### 3.2.2 Interaction force characteristics

This metric includes the maximum and average forces exerted by the participant during reciprocal movements, as well as the time-varying force profile. It is used to assess the impact of different control strategies on the user’s effort requirements, specifically whether the assistance appropriately reduces the physical load or results in excessive support.

#### 3.2.3 Training duration and repetition count

This metric records the actual duration of each training session and the number of effective movement repetitions completed by the participant. It directly reflects the improvement in training endurance.

### 3.3 Comparative experiments

**FIGURE 5:**
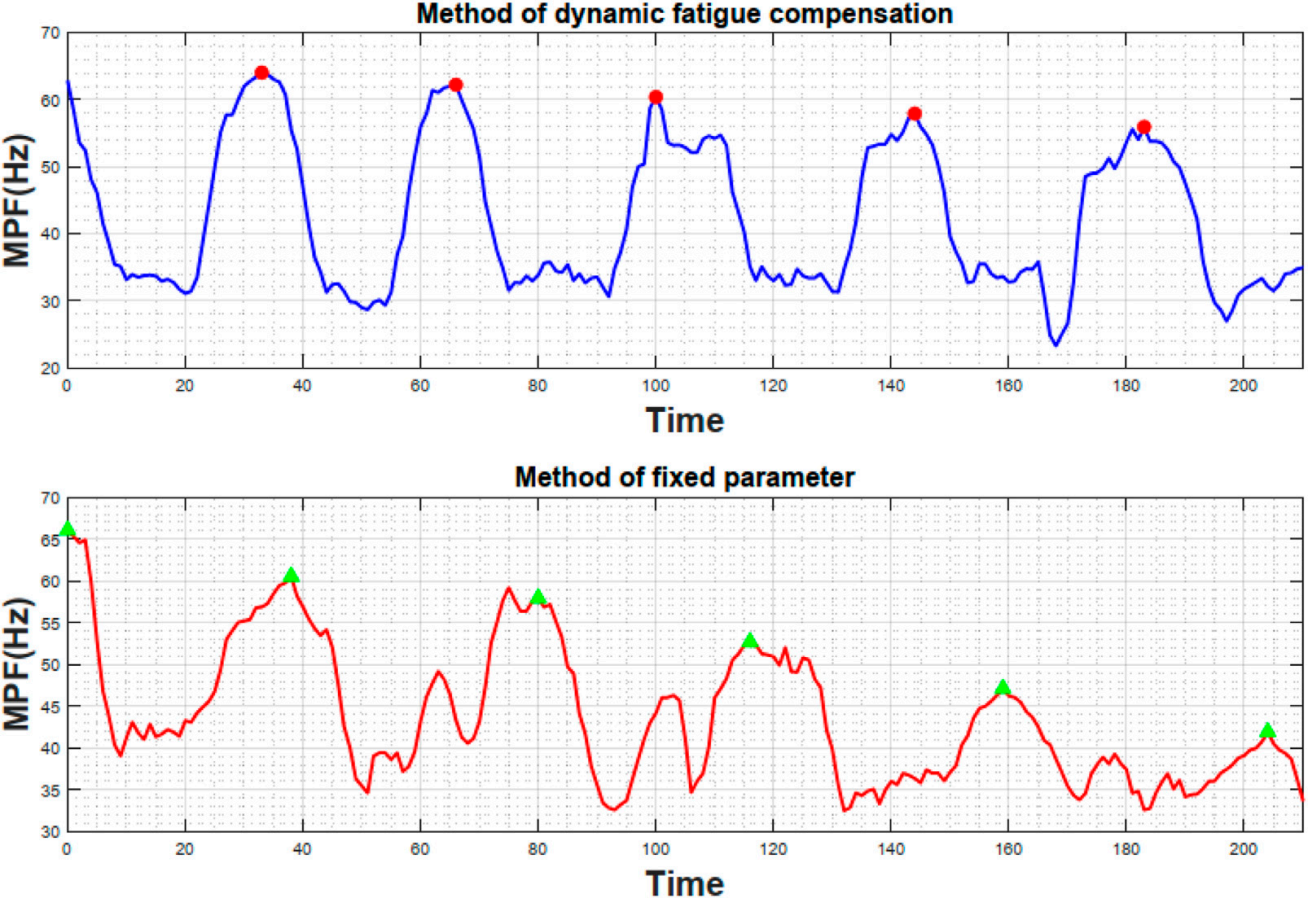
Comparison of MPF variation under different control strategies.

**FIGURE 6:**
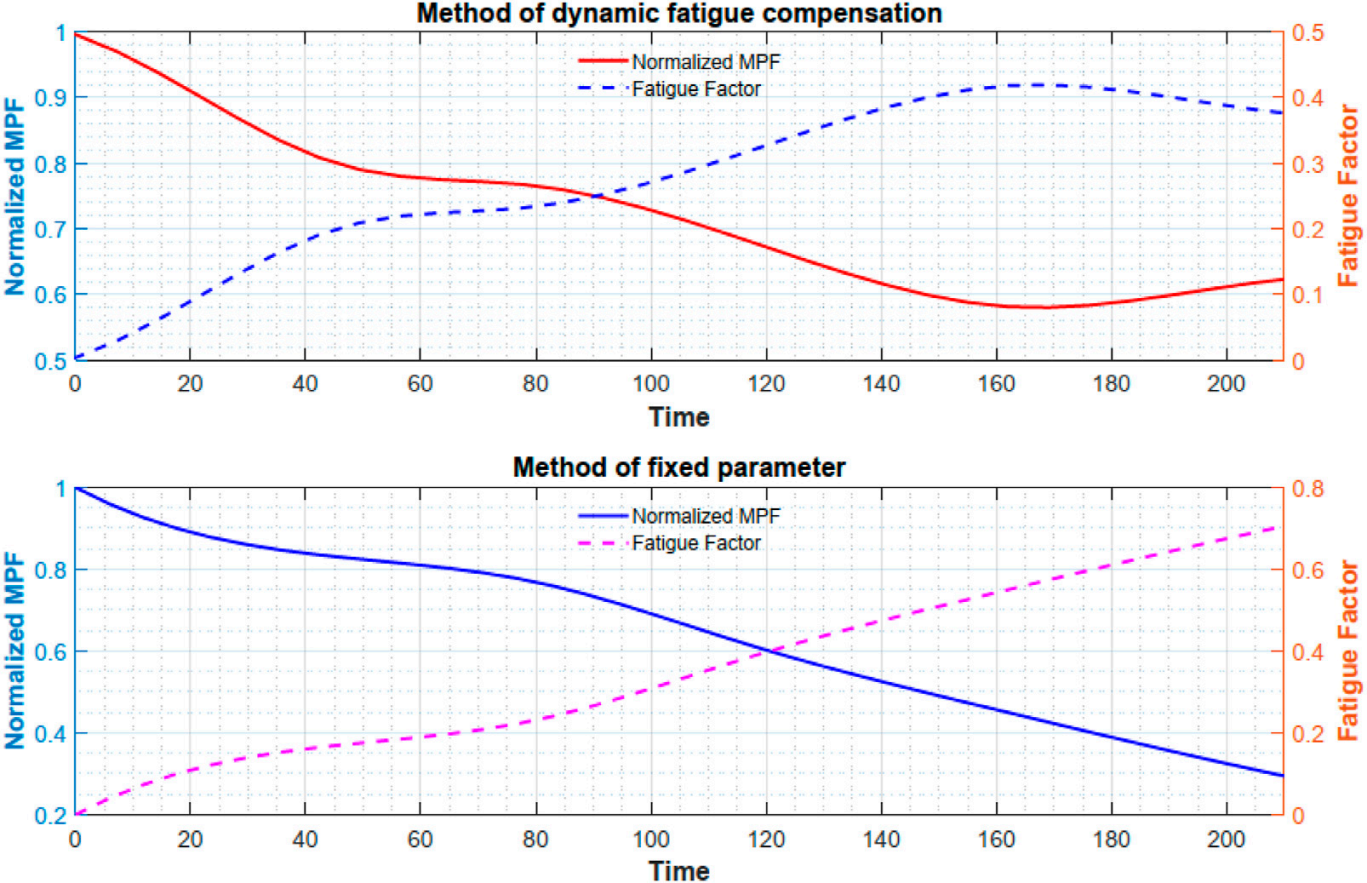
Comparison of normalized MPF and fatigue factor under different control methods.

**FIGURE 7:**
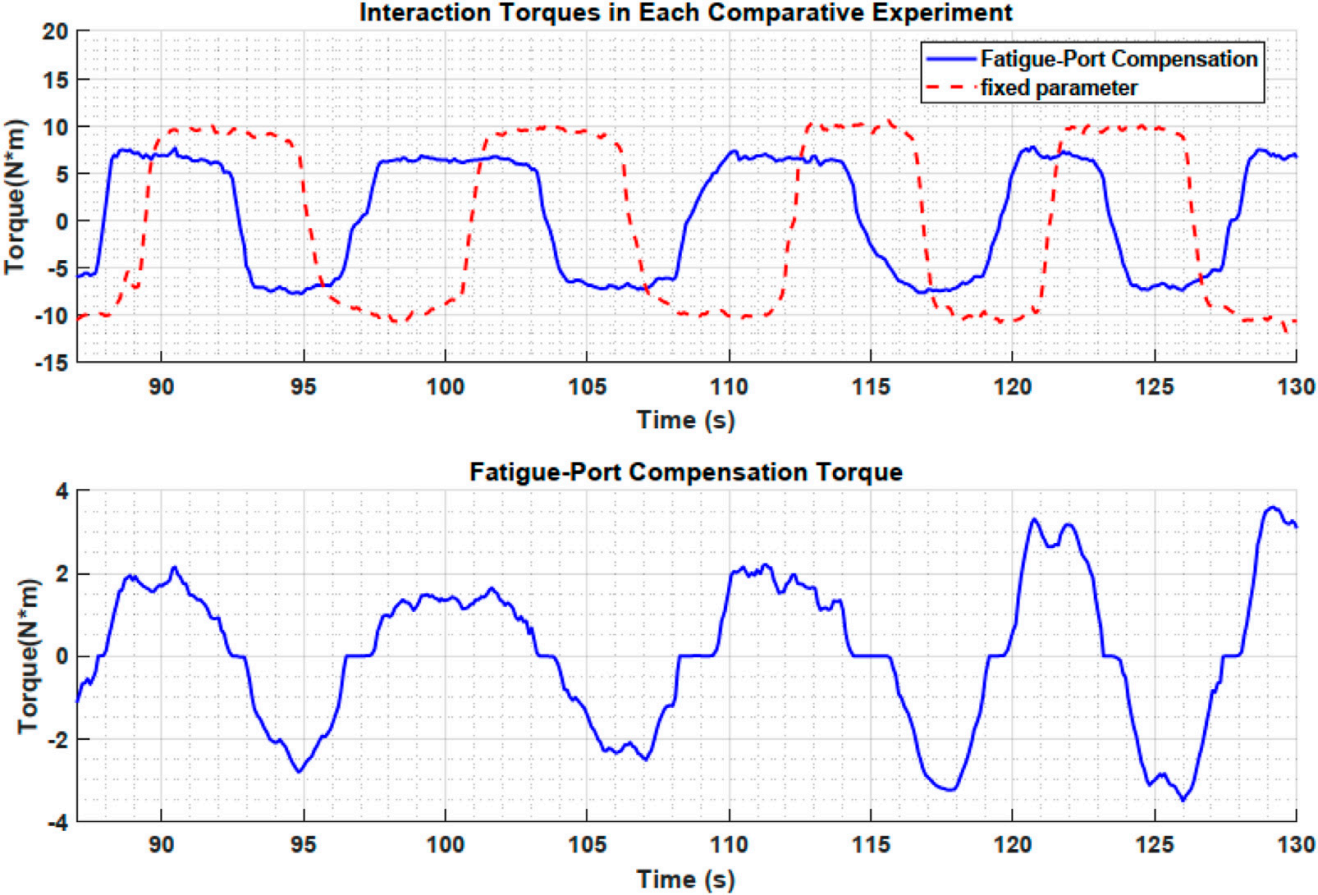
Interaction torques under different control methods (top) and corresponding fatigue-port compensation torque (bottom).

Figures 5–7 illustrate the time-varying changes in EMG signals, fatigue factors, human-robot interaction force, and the robot’s fatigue-dissipation port compensation force for a typical subject under two different control strategies. The different control methods result in varying growth trends of muscle activation level (EMG signals) and fatigue factors, while interaction force and compensation force also adjust dynamically. These findings not only underscore the critical importance of the fatigue factor in closed-loop control but further demonstrate the effectiveness of the fatigue-dissipation port compensation strategy in rehabilitation training. The following section provides a more detailed analysis and discussion based on the specific data and trends shown in the figures.

Figure 5 presents the raw surface electromyography (sEMG) signals recorded via the arm band. From the signal peaks, it is evident that the proposed fatigue dissipation port compensation method yields a slower decline in the mean power frequency (MPF) of the sEMG signals. Following data processing and in accordance with Formula 8; Figure 6 is obtained. Figure 6 illustrates the variation of the biceps brachii muscle fatigue index over the training duration under two control strategies, where the magenta dashed line represents the fixed-parameter control (A), and the blue dashed line denotes the fatigue-compensation control (B). Under fixed-parameter control, the muscle fatigue index rises nearly linearly from zero and reaches about 0.47 at around 140 s. By contrast, when using the proposed method, although the initial rate of increase is similar to that of the fixed-parameter control, compensation via the fatigue port is activated as fatigue intensifies, significantly decelerating the rate of fatigue buildup. Consequently, at 140 s, the blue dashed line only reaches approximately 0.37 and then enters a plateau, indicating saturation rather than continued rapid escalation. A quantitative assessment shows that under fixed damping parameters, the fatigue growth rate is about 0.2, whereas under the proposed method it falls to around 0.11 in the latter stage, representing 45%. These findings demonstrate that incorporating fatigue modeling into the control strategy effectively mitigates the accumulation rate of muscle fatigue.

Furthermore, there is a clear difference in training duration between the two control strategies. Under fixed-parameter control, participants typically reach exhaustion at around 240 ±20 s on average. By contrast, with the proposed method, the average training duration extends to 400 ±20 s, which represents around 40% increase compared to the fixed-parameter approach. Consequently, participants also achieve a higher number of exercise repetitions. These findings indicate that incorporating the dynamic behavior of the fatigue dissipation port into the compensatory control strategy effectively prolongs the duration of productive training.

Figure 7 compares the time-varying human-robot interaction forces under different control strategies. Because the total training duration is relatively long, only the segment in which the two methods differ most significantly is shown here to more clearly demonstrate the advantages of the proposed approach. Taking elbow flexion primarily driven by the biceps brachii as an example, the fixed-parameter control strategy requires approximately 10 N m of torque for each flexion. Participants can maintain this effort level for the first few minutes, but as fatigue accumulates, their movement speed gradually declines even though they attempt to sustain the same force output. This trend can be observed both in the number of peak values in Figure 1 and in the time required for each flexion-extension cycle in Figure 7.

In contrast, under the proposed method, the compensatory force provided by the fatigue port is relatively small during the early stage when fatigue is mild, so the interaction force does not differ substantially from that of the fixed-parameter control. However, as fatigue increases, the proposed method partially takes over the load through the fatigue port, reducing the average force required from the user. As shown in Figure 7, the user’s exerted force decreases significantly in the later phase, mitigating further fatigue accumulation while maintaining the necessary movement speed. Meanwhile, the user still must provide a certain level of force to participate in the training, ensuring that the robot does not completely take over and that sufficient training intensity is preserved for rehabilitation. According to participant feedback, their subjective sense of exertion in the latter phase is notably lower compared to the fixed-parameter mode, yet they can still feel their muscles continuously engaged rather than entirely relaxed. This dynamic assistance mechanism effectively balances training intensity and endurance, extends the duration of effective training, and enhances user comfort and cooperation.

Overall, the experimental results strongly validate the effectiveness of the proposed method. By incorporating dynamic modeling of muscle fatigue, the rehabilitation robot demonstrated significantly improved training continuity and adaptability. This finding is consistent with conclusions reported in related studies (Mashayekhi and Moghaddam, 2022), which suggest that adaptive training based on electromyographic fatigue feedback can effectively extend the duration of high-intensity exercise and substantially increase the number of movement repetitions. Furthermore, the proposed method enhances the smoothness and safety of human-robot interaction at the control level, ensuring that the training system maintains stable and reliable performance across varying fatigue states.

## 4 Discussion

This study proposes and validates a novel control strategy to address the issue of muscle fatigue during prolonged training with upper-limb rehabilitation robots. Before discussing the practical implications of this approach, we first examine its key advantages and potential limitations when compared to the fixed damping parameter control method.

### 4.1 Advantages

Conventional fixed-parameter control strategies are limited in their ability to adapt to the user’s real-time physiological state, often leading to training interruption or decreased training quality during later stages due to muscular fatigue. The control method proposed in this study utilizes surface electromyography (sEMG) to continuously assess muscle fatigue levels and adaptively adjusts assistance accordingly. This enables patients to complete the intended training movements even when approaching fatigue limits, thereby ensuring the integrity of the training “dosage.” Previous studies (Mashayekhi and Moghaddam, 2022) have also indicated that dynamically adjusting training intensity based on fatigue indicators can significantly extend the duration of effective training. The experimental results presented in this study further validate this finding, showing that the introduction of fatigue compensation control leads to an average increase of over 50% in both training duration and repetition count. In the context of neurorehabilitation, the frequency and repetition of training are critical for promoting neural plasticity and functional recovery. Therefore, this method achieves high-intensity, high-frequency “high-dosage” training without increasing patient risk, demonstrating considerable clinical potential.

At the same time, fatigue during training often leads to decreased motor coordination and, in severe cases, compensatory movements or involuntary exertion, which increase the risk of injury. To address these issues, this study employs the Port-Hamiltonian Systems (PHS) framework, incorporating all control actions within a unified energy conservation and dissipation analysis structure to theoretically guarantee passivity and stability of the system. Given that rehabilitation patients are typically physically vulnerable, intrinsic system stability and sufficient safety margins are critical for clinical applications. The introduction of the PHS framework not only enhances the robustness of the control system in dynamic conditions but also fundamentally reinforces the inherent safety of the human-robot system, providing a more reliable and secure foundation for rehabilitation training.

Additionally, the method proposed in this study has high scalability. While the muscle fatigue estimation method is primarily applied to upper-limb rehabilitation, especially through monitoring the EMG signals of the biceps and triceps, the decrease in MPF with muscle fatigue is a widely recognized physiological phenomenon. Therefore, this method can be extended to fatigue assessment in other body parts, such as lower limbs or trunk muscles. For different muscle groups, the method can be appropriately adjusted based on the physiological characteristics of the target muscle group, the quality of EMG signals, and the differences in training tasks. This includes modifications in signal preprocessing techniques and parameter settings, ensuring its broad applicability.

### 4.2 Limitations and challenges

The effectiveness of the proposed method has been validated in single-joint flexion-extension tasks. Future work will focus on extending its application to more complex multi-degree-of-freedom rehabilitation robots, such as those involving coordinated shoulder-elbow movements or lower limb training. These systems involve a greater number of muscle groups and more complex dynamics, posing significant challenges for fatigue modeling and control strategy design, which require further in-depth investigation to enhance the generalizability and adaptability of the method.

Moreover, although the proposed approach has demonstrated performance advantages in laboratory experiments with healthy subjects, the ultimate evaluation criterion for rehabilitation robots lies in their clinical effectiveness, specifically their ability to promote functional recovery in patients. Therefore, future research will involve close collaboration with rehabilitation medicine experts to conduct systematic clinical trials in target patient populations. These studies will assess the applicability and therapeutic efficacy of the method across different injury types and stages of recovery, aiming to facilitate its translation into real-world clinical practice.

## 5 Conclusion

This paper proposes a control method incorporating muscle fatigue dynamics to address the issue of muscle fatigue during upper limb rehabilitation robot training. A dual-loop control architecture is developed based on the port-Hamiltonian system (PHS) theory. Within the PHS framework, a muscle fatigue dynamic model is established by treating muscle fatigue as a time-varying joint damping effect. The degree of fatigue is estimated in real-time using the median frequency of sEMG signals, and a fatigue index is defined accordingly. Time-varying damping is introduced into the port-Hamiltonian model, enabling explicit modeling and quantitative description of fatigue effects in the human body, thus providing an adjustable fatigue-related parameter for control. Based on this model, a fatigue compensation control strategy is designed. The outer loop employs admittance control to regulate the desired trajectory, while the inner loop, implemented within the Hamiltonian framework, achieves trajectory tracking through energy shaping and damping injection. This approach compensates for fatigue-induced dynamic changes and maintains closed-loop passive stability.

Comparative experiments conducted on healthy subjects have demonstrated that, compared to fixed-parameter control, the proposed control strategy incorporating fatigue modeling significantly reduces the rate of muscle fatigue accumulation (by approximately 45%), thereby extending the duration and number of repetitions of training sessions (by approximately 40%). At the same time, since the port-Hamiltonian passive control ensures energy regulation throughout the training process, no unstable oscillations or hazardous movements were observed during the experiments. When subjects experienced severe fatigue, the robot was able to automatically transition to an assistive mode, thereby preventing training interruptions and reducing the risk of injury. Conversely, when sufficient muscle strength was detected, appropriate resistance was provided by the robot to ensure active participation in training. This human-robot interaction mechanism offers clear advantages in enhancing training effectiveness while preventing excessive fatigue.

## Data Availability

The original contributions presented in the study are included in the article/Supplementary Material, further inquiries can be directed to the corresponding authors.

## References

[B1] Ai Q. Liu Z. Meng W. Liu Q. Xie S. Q. (2023). Uncertainty compensated high-order adaptive iteration learning control for robot-assisted upper limb rehabilitation. IEEE Trans. Automation Sci. Eng. 21 (4), 7004–7015. 10.1109/TASE.2023.3335401

[B2] Cai S. Xie P. Li G. Xie L. (2024). Compensation-corrective adaptive control strategy for upper-limb rehabilitation robots. Robotics Aut. Syst. 177, 104701. 10.1016/j.robot.2024.104701

[B3] Fujimoto K. Sakata N. Maruta I. Ferguson J. (2020). A passivity-based sliding mode controller for simple port-Hamiltonian systems. IEEE Control Syst. Lett. 5 (3), 839–844. 10.1109/LCSYS.2020.3005327

[B4] Ghajari S. Moghaddam R. K. Kobravi H. Pariz N. (2023). Muscle fatigue regulation through muscle activation control in a knee hybrid exoskeleton: simulation study. Machines 11 (10), 937. 10.3390/machines11100937

[B5] Groothuis S. S. Folkertsma G. A. Stramigioli S. (2018). A general approach to achieving stability and safe behavior in distributed robotic architectures. Front. Robotics AI 5, 108. 10.3389/frobt.2018.00108/frobt.2018.00108 PMC7806010 33500987

[B6] Groothuis S. S. Stramigioli S. Carloni R. (2017). Modeling robotic manipulators powered by variable stiffness actuators: a graph-theoretic and port-Hamiltonian formalism. IEEE Trans. Robotics 33 (4), 807–818. 10.1109/TRO.2017.2668385

[B7] Kim S.-K. Kim Y. Ahn C. K. (2021). Energy-shaping speed controller with time-varying damping injection for permanent-magnet synchronous motors. IEEE Trans. Circuits Syst. II Express Briefs 68 (1), 381–385. 10.1109/TCSII.2020.2992260

[B8] Lai Y. Sutjipto S. Clout M. D. Carmichael M. G. Paul G. (2018). GAVRe2: towards data-driven upper-limb rehabilitation with adaptive-feedback gamification. IEEE ROBIO, 164–169. 10.1109/ROBIO.2018.8665105

[B9] Li J. Li G. Chen Z. Li J. (2022). A novel EMG-based variable impedance control method for a tele-operation system under an unstructured environment. IEEE Access 10, 89509–89518. 10.1109/ACCESS.2022.3200696

[B10] Liu C. Zhao K. Si W. Li J. Yang C. (2025). Neuroadaptive admittance control for human-robot interaction with human motion intention estimation and output error constraint. IEEE Trans. Cybern., 1–12. 10.1109/TCYB.2025.3555104 40198290

[B11] Lynch K. M. Park F. C. (2021). Modern robotics: mechanics, planning, and control. Cambridge University Press.

[B12] Mahfouz D. M. Shehata O. M. Morgan E. I. Arrichiello F. (2024). A comprehensive review of control challenges and methods in end-effector upper-limb rehabilitation robots. Robotics 13 (12), 181. 10.3390/robotics13120181

[B13] Mashayekhi M. Moghaddam M. M. (2022). EMG-driven fatigue-based self-adapting admittance control of a hand rehabilitation robot. J. Biomechanics 138, 111104. 10.1016/j.jbiomech.2022.111104 35561557

[B14] Rashad R. Bicego D. Zult J. Sanchez-Escalonilla S. Jiao R. Franchi A. (2022). Energy aware impedance control of a flying end-effector in the port-Hamiltonian framework. IEEE Trans. Robotics (TRO) 38 (6), 3936–3955. 10.1109/tro.2022.31835322022.3183532

[B15] Rashad R. Califano F. Stramigioli S. (2019). Port-Hamiltonian passivity-based control on SE(3) of a fully actuated UAV for aerial physical interaction near-hovering. IEEE Robotics Automation Lett. 4 (4), 4378–4385. 10.1109/lra.2019.2932864

[B16] Sakata N. Fujimoto K. Maruta I. (2024). Passivity-based sliding mode control for mechanical port-Hamiltonian systems. IEEE Trans. Automatic Control 69 (8), 5605–5612. 10.1109/TAC.2024.3371898

[B17] Sandoval J. Kelly R. Santibáñez V. Moreno-Valenzuela J. Cervantes-Pérez L. (2024). Partial potential energy shaping control of torque-driven robot manipulators in joint space. Int. J. Control Automation Syst. 22 (7), 2230–2241. 10.1007/s12555-022-1196-z

[B18] Thacham Poyil A. Steuber V. Amirabdollahian F. (2020). Adaptive robot mediated upper limb training using electromyogram-based muscle fatigue indicators. PLoS ONE 15 (5), e0233545. 10.1371/journal.pone.0233545pone.0233545 32469912 PMC7259541

[B19] Tian D. Li F. He Y. Li W. Chen Z. Yang M. (2024). Data-driven estimation for uphill continuous rehabilitation motion at different slopes using sEMG. Biomed. Signal Process. Control 93, 106162. 10.1016/j.bspc.2024.106162

[B20] Tian D. Li W. Li J. Li F. Chen Z. He Y. (2024). Self-balancing exoskeleton robots designed to facilitate multiple rehabilitation training movements. IEEE Trans. Neural Syst. Rehabilitation Eng. 32, 293–303. 10.1109/TNSRE.2023.3348985 38163311

[B21] Vafadar A. K. Côté J. N. Archambault P. S. (2012). The effect of muscle fatigue on position sense in an upper limb multi-joint task. Mot. Control 16 (2), 265–283. 10.1123/MCJ.16.2.265 22357216

[B22] Wan J. J. Qin Z. Wang P. Y. Sun Y. Liu X. (2017). Muscle fatigue: general understanding and treatment. Exp. Mol. Med. 6 (49), e384. 10.1038/emm.2017.194 PMC5668469 28983090

[B23] Xu L. Yan Y. Wang W. Su T. He G. Li G. (2024). Adaptive human–robot interaction torque estimation with high accuracy and strong tracking ability for a lower limb rehabilitation robot. IEEE/ASME Trans. Mechatronics 29 (6), 4814–4825. 10.1109/TMECH.2024.3394491

[B24] Zhang Y.-P. Cao G.-Z. Li L.-L. Diao D.-F. (2024). Interactive control of lower limb exoskeleton robots: a review. IEEE Sensors J. 24 (5), 5759–5784. 10.1109/JSEN.2024.3352005

[B25] Zhou J. Li Z. Li X. Wang X. Song R. (2021). Human-robot cooperation control based on trajectory deformation algorithm for a lower limb rehabilitation robot. IEEE/ASME Trans. Mechatronics 26 (6), 3128–3138. 10.1109/TMECH.2021.3053562

