# Supplementary figures and images for: Research on rehabilitation robot control based on port-Hamiltonian systems and fatigue dissipation port compensation
Source: Front Bioeng Biotechnol. 2025 May 23;13:1609548. doi: 10.3389/fbioe.2025.1609548 (PMC12141283; doi:10.3389/fbioe.2025.1609548)

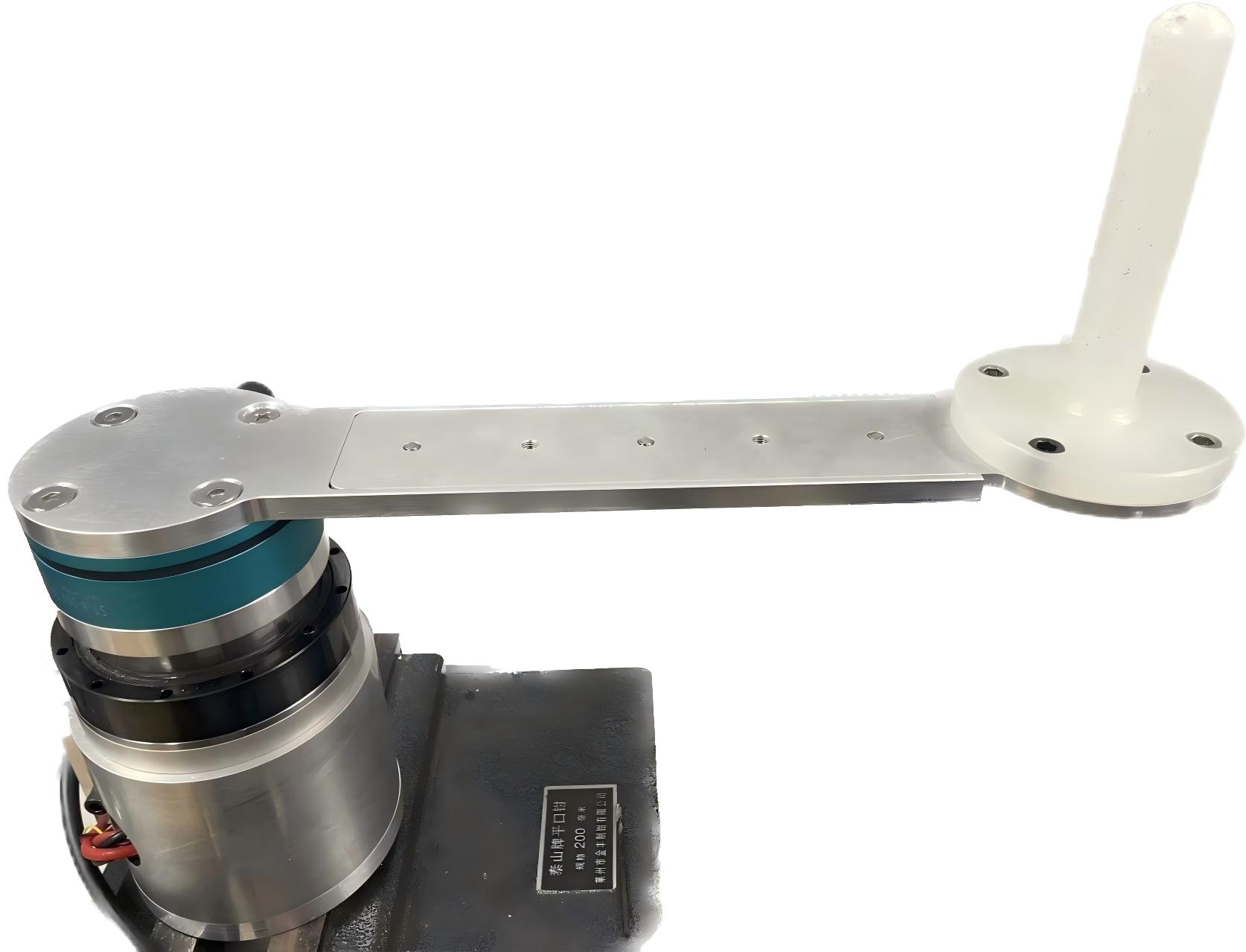

Supplement: Supplementary file 1 [file Image5.jpeg]

**Method of dynamic fatigue compensation**

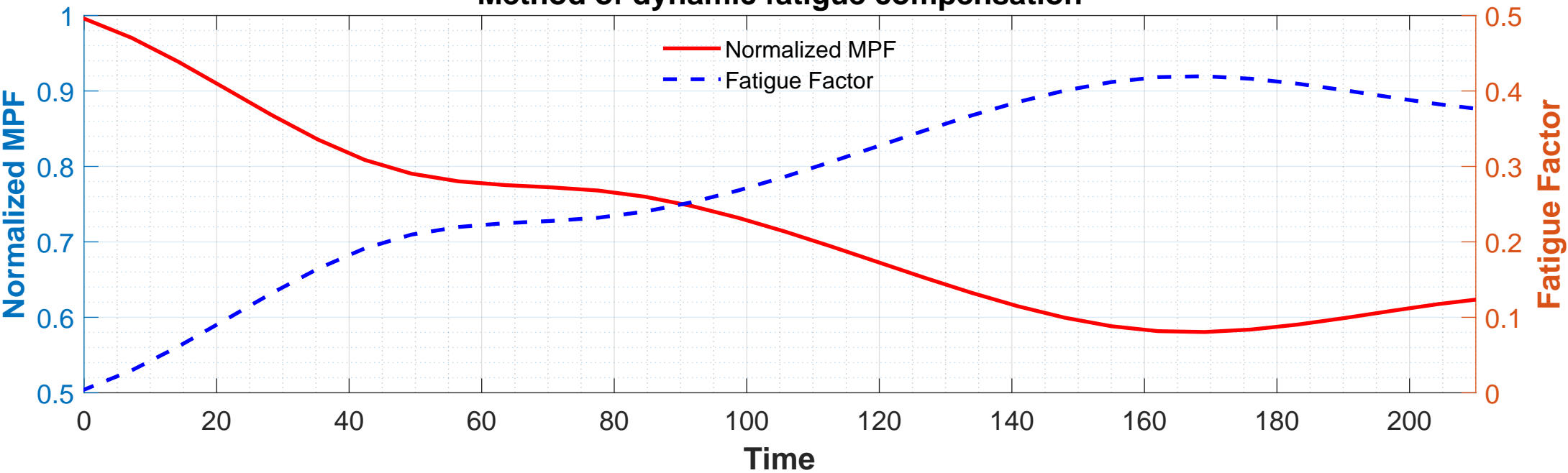

**Method of fixed parameter**

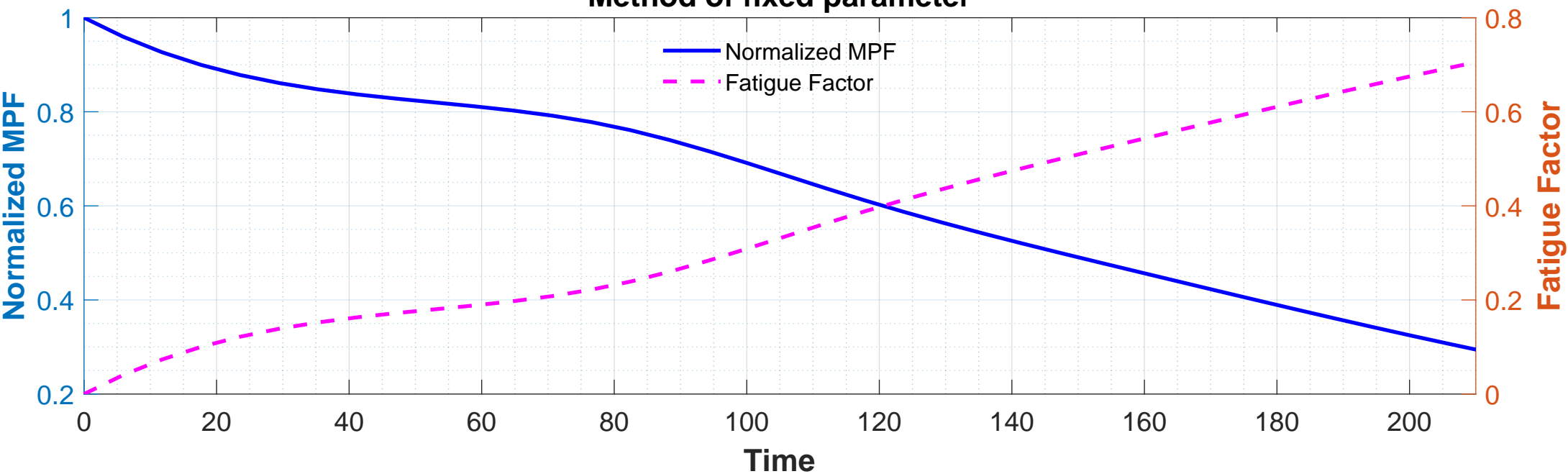

Supplement: Supplementary file 2 [file Image4.pdf]

**Interaction Torques in Each Comparative Experiment**

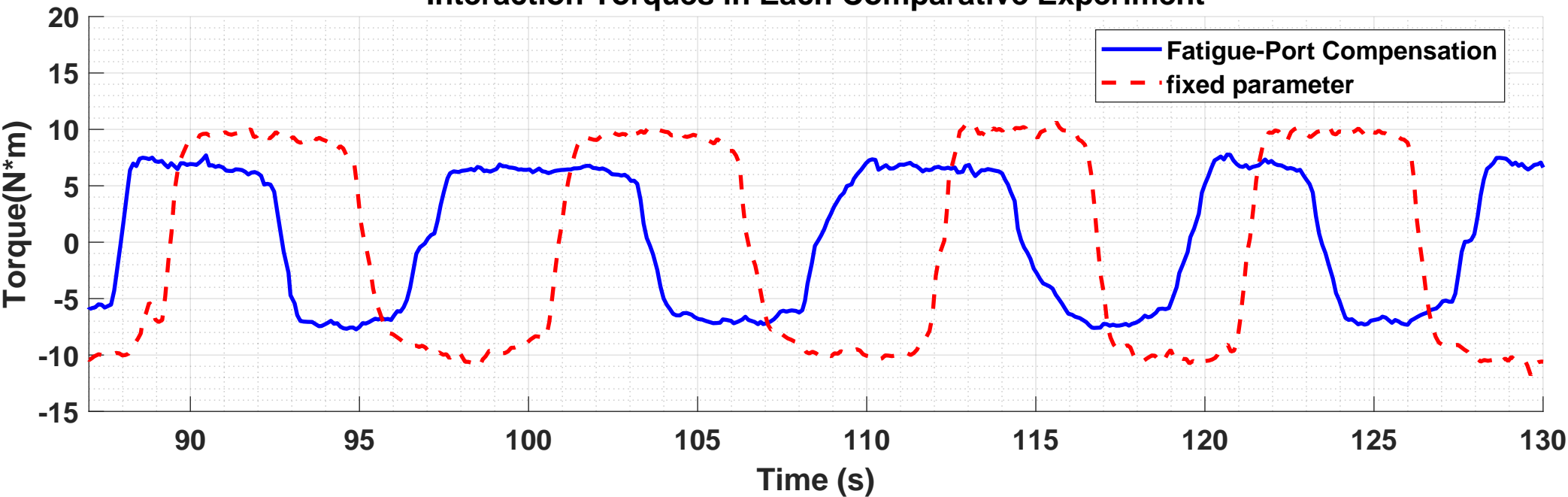

**Fatigue-Port Compensation Torque**

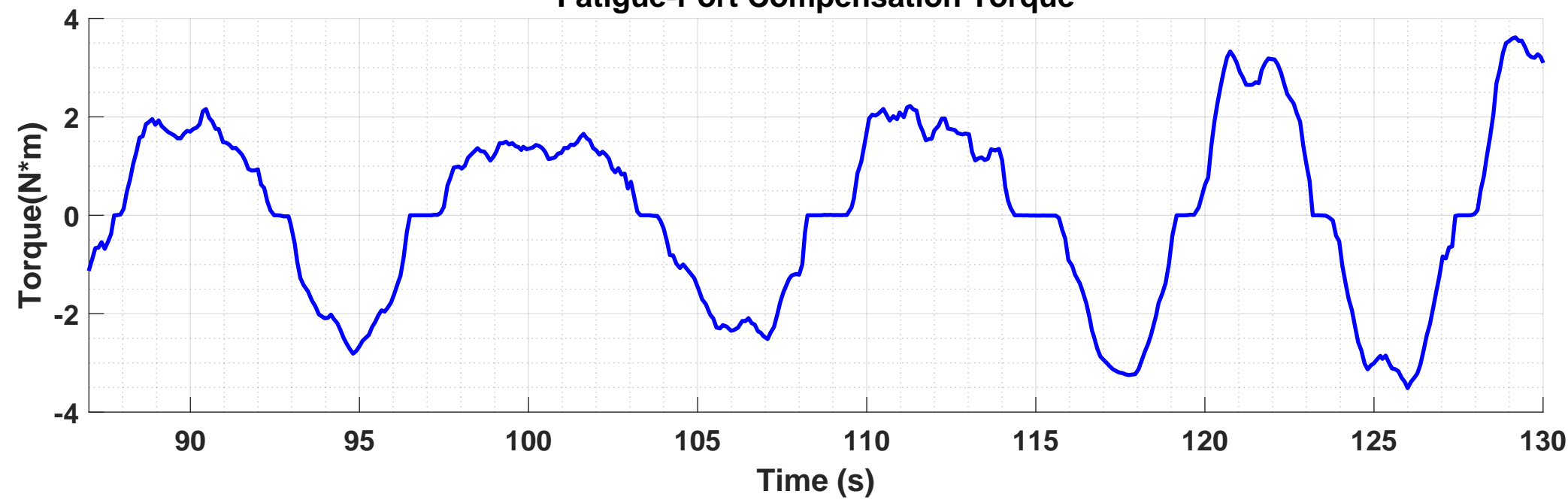

Supplement: Supplementary file 3 [file Image2.pdf]

**Method of dynamic fatigue compensation**

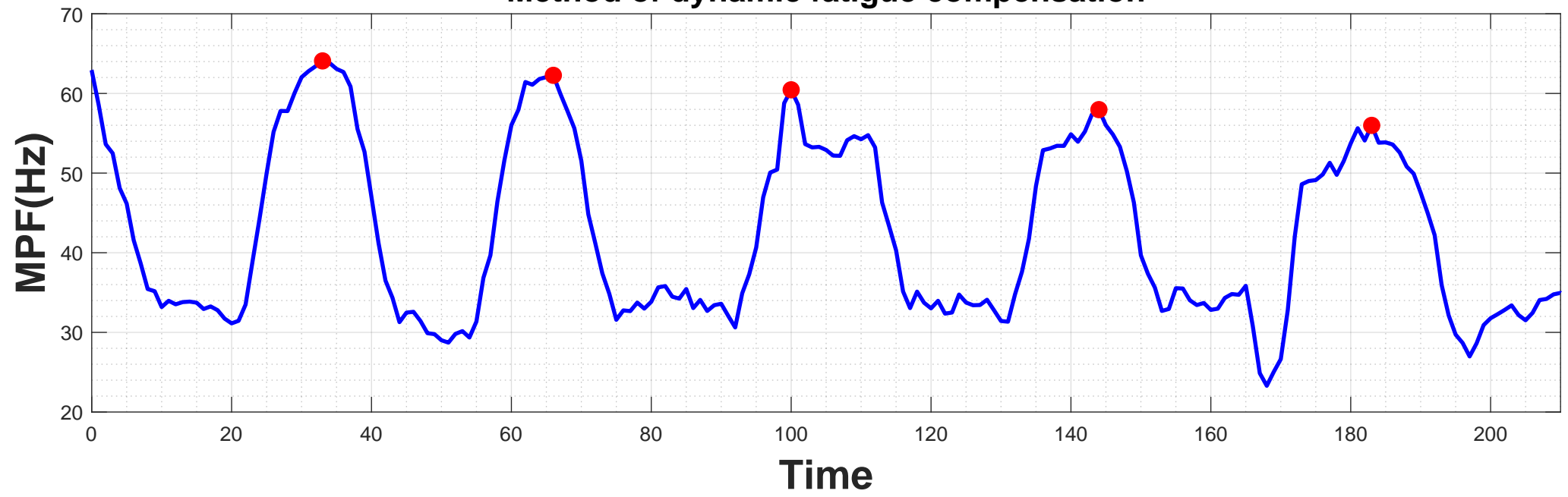

**Method of fixed parameter**

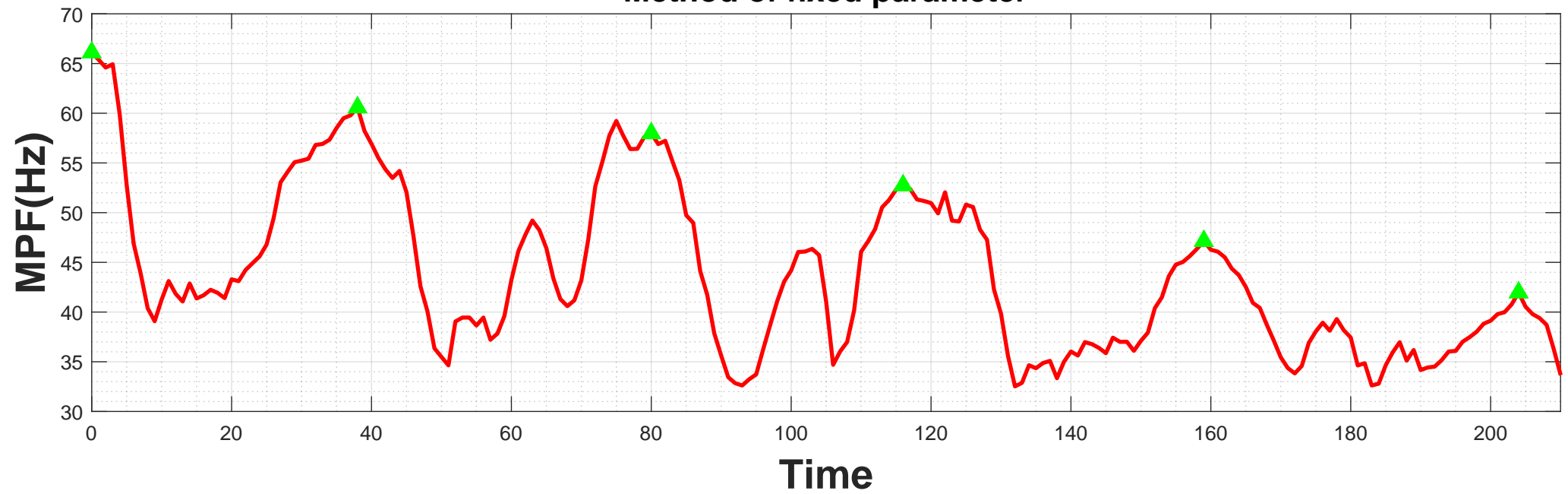

Supplement: Supplementary file 4 [file Image3.pdf]

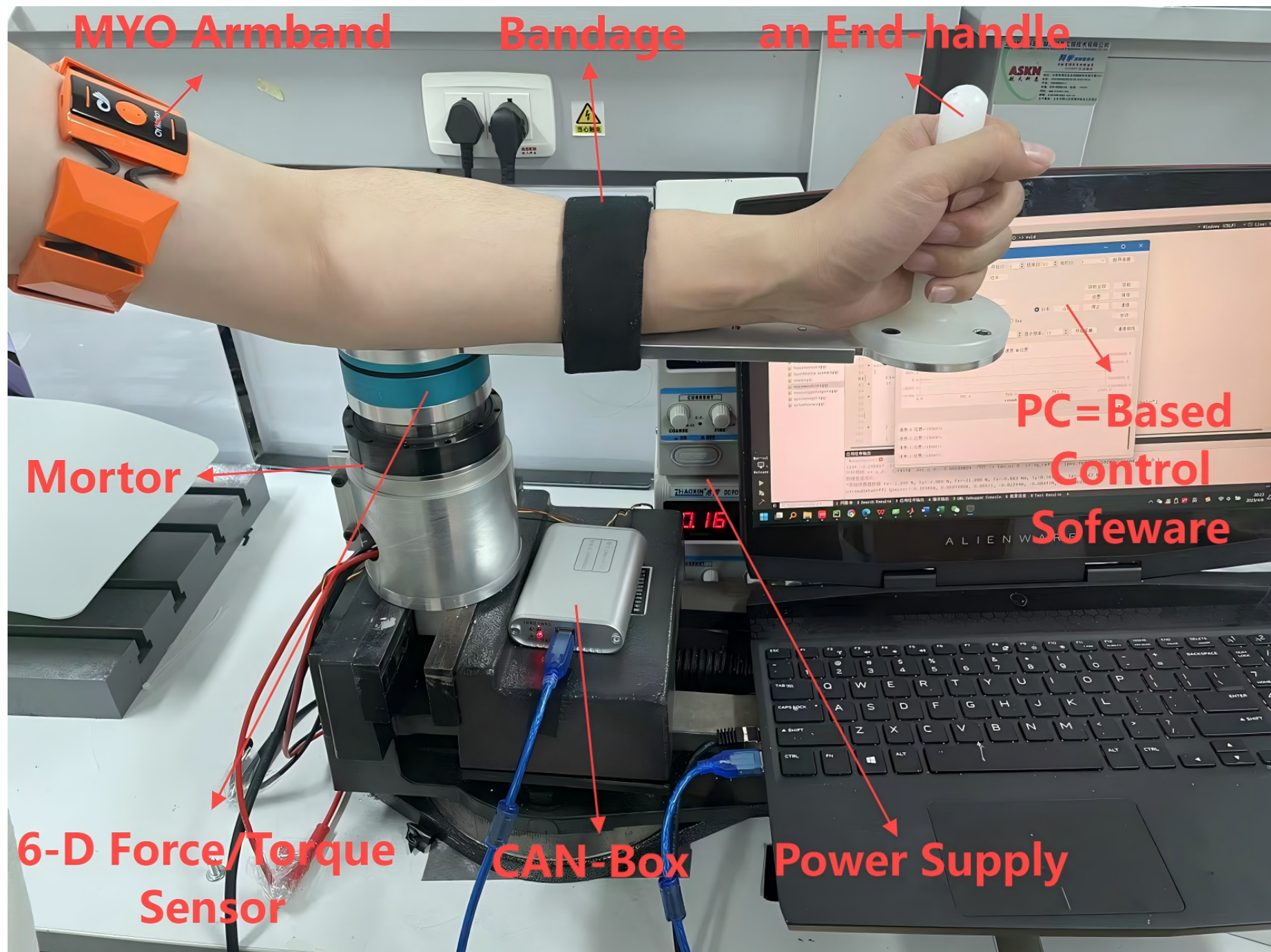

Supplement: Supplementary file 5 [file Image1.pdf]

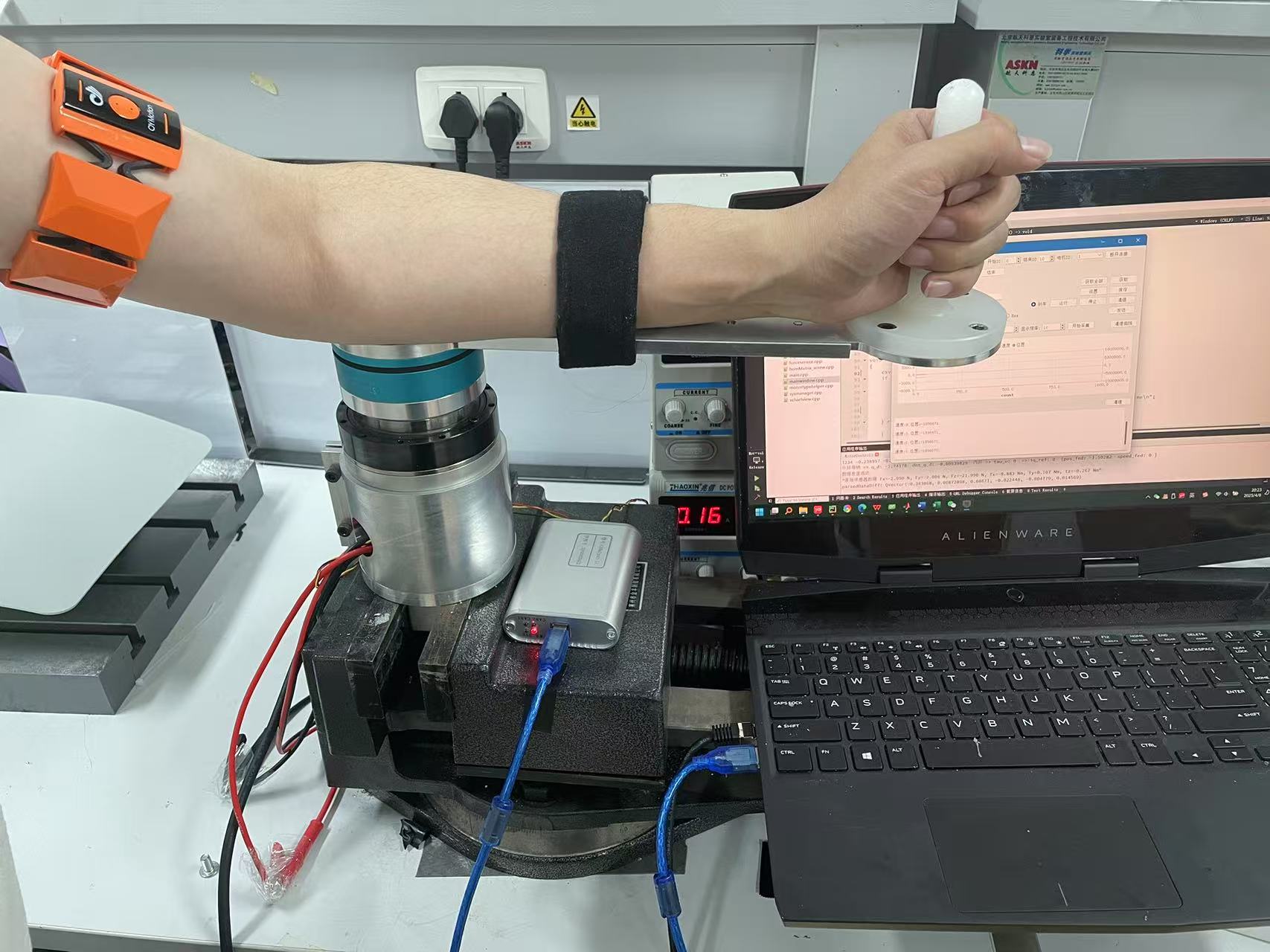

Supplement: Supplementary file 6 [file Image6.jpeg]
